# Sequence analysis of integrated hepatitis B virus DNA during HBeAg-seroconversion

**DOI:** 10.1038/s41426-018-0145-7

**Published:** 2018-08-08

**Authors:** Magdalena Agnieszka Budzinska, Nicholas Adam Shackel, Stephan Urban, Thomas Tu

**Affiliations:** 1 Centenary Institute, University of Sydney, Sydney, 2050 NSW Australia; 2 South Western Sydney Clinical School, University of New South Wales, Sydney, 2170 NSW Australia; 3 Liverpool Hospital, Gastroenterology, Sydney, 2170 NSW Australia; 4 Department of Infectious Diseases, Molecular Virology, Heidelberg University Hospital, Heidelberg, D-69120 Germany; 5 German Center for Infection Research (DZIF), Partner Site Heidelberg, Heidelberg, D-69120 Germany

## Abstract

Hepatitis B virus (HBV) integration into the host cell genome occurs early on in infection and reportedly induces pro-oncogenic changes in hepatocytes that drive HCC initiation. However, it remains unclear when these changes occur during hepatocarcinogenesis. Extensive expansion of hepatocyte clones with a selective advantage was shown to occur prior to cancer formation during the HBeAg-seroconversion phase of chronic HBV infection. We hypothesized that since integrations occur during the early stages of infection, cell phenotype could be altered and induce a selection advantage (e.g., through insertional mutagenesis or *cis*-mediated activation of downstream genes). Here, we analyzed the enrichment of genomic and functional patterns in the cellular host sequence adjacent to HBV DNA integration events. We examined 717 unique integration events detected in patients who have and have not undergone HBeAg-seroconversion (*n* = 41) or in an in vitro model system. We also used an in silico model to control for detection biases. We showed that the sites of HBV DNA integration were distributed throughout the entire host genome without obvious enrichment of specific structural or functional genomic features in the adjacent cellular genome during HBeAg-seroconversion. Currently, this is the most comprehensive characterization of HBV DNA integration events prior to hepatocarcinogenesis. Our results suggest no significant selection for (or against) specific cellular sites of HBV DNA integration occur during the clonal expansion phase of chronic HBV infection. Thus, HBV DNA integration events likely represent passenger events rather than active drivers of liver cancer, which was previously suggested.

## Introduction

Chronic infection with hepatitis B virus (HBV) is one of the most widespread causes of liver cirrhosis and primary liver cancer (in the form of hepatocellular carcinoma; HCC). Chronic HBV infection is currently incurable, affects ~240 million people worldwide, and is the main contributor towards viral hepatitis-associated morbidity and mortality^1^. HBV-associated disease progression is largely driven by persistence of HBV infection and the resultant chronic anti-viral inflammatory response^2,3^. In addition, HCC initiation is reportedly driven by the integration of HBV DNA into the host cell genome via direct mutations in cancer-associated genes^4–6^, chromosomal instability^7,8^, *cis*-activation of cellular genes^5–7,9^, and the persistent expression of mutant HBV proteins that drive cellular stress^10^.

Integrated HBV DNA is a replication-deficient form of the virus that is generated as a by-product of HBV viral replication^11^. Following receptor-mediated entry, the HBV nucleocapsid containing the relaxed-circular DNA (rcDNA) or more rarely, the double-stranded linear DNA (dslDNA), HBV genome is released into the cytoplasm and transported to the nucleus^12^. Intranuclear HBV DNA is converted into covalently closed circular DNA (cccDNA), which is the stable episomal transcriptional template for HBV mRNAs. An additional possible fate for intra-nuclear dslDNA (contained within either the input virus or possibly those newly generated by the infected cell) HBV genomes is integration into the host cell genome at the site of double-stranded DNA breaks by non-homologous end joining (NHEJ) or in some instances, microhomology mediated end-joining (MMEJ)^13,14^. Integration is observed at a frequency of 1 in ~10^4^ cells in cell culture infection systems^14^, in woodchuck and duck models of HBV infections^15,16^ and in chronically infected HBV patients^17–19^.

HBV DNA integration can be detected in all stages of chronic HBV infection. In the initial low inflammation phase of chronic HBV infection (generally the first three decades of a lifelong chronic infection), large numbers of small hepatocyte clones that contain integrated HBV DNA are detectable^19^. During this phase, high levels of secreted viral antigens, particularly HBV surface and e antigens (HBsAg and HBeAg, respectively), can be detected in the blood.

Upon the immune activation phase of hepatitis B infection, there is a strong anti-viral immune response against HBV-expressing hepatocytes. After many (but not all) of the HBV-expressing hepatocytes are cleared and replaced by compensatory mitosis of surrounding hepatocytes, both serum HBV antigens and HBV DNA titers are reduced. The clinical marker for this phase is the neutralization of serum HBeAg (which decreases due to the immune-mediated killing of HBV-expressing hepatocytes and the rising incidence of HBV mutants that express a low amount of HBeAg due to basal core promoter and pre-core mutations) by an excess of circulating anti-HBeAg antibodies.

During the process of HBeAg-seroconversion and its associated liver turnover, hepatocytes with integrated HBV DNA undergo selective clonal expansion, allowing for natural selection and evolutionary bottlenecks. Clonal expansion of histologically normal hepatocytes is associated with HBeAg-seroconversion; hepatocyte clones of >10,000 cells have been observed in HBeAg-negative patients, which is a >10-fold increase in clone size compared to HBeAg-positive patients^18,19^. Mathematical simulations have shown that hepatocyte clones of this size were unlikely to have formed due to random liver turnover, but instead likely represented expansion of hepatocytes with a selection advantage^18^. These data support a “field cancerization” model, wherein histologically normal cells with pre-neoplastic changes that convey a selective advantage are allowed to proliferate (e.g., due to chronic inflammation), thereby dramatically increasing the probability of a cell clone accumulating subsequent cancer driver mutations^2^. Indeed, clonal expansion of histologically normal cells is a risk factor for carcinogenesis in other gastrointestinal cancers, including esophageal and colorectal cancer^20,21^.

Previous reports have suggested that HBV DNA integration induces changes in hepatocytes that drive HCC initiation via insertional mutagenesis^4–6^ or *cis*-regulation of cellular genes^5–7,9^. We reasoned that if HBV DNA integration is involved in the early stages of carcinogenesis, then specific subsets of integrations should be enriched during the strong clonal expansion associated with HBeAg-seroconversion. Therefore, we analyzed functional patterns with respect to the cellular host sequence close to HBV DNA integration junctions detected in HBeAg-positive and HBeAg-negative HBV patients.

## Results

### Detection of HBV DNA integration and controls for detection bias

**Fig. 1:**
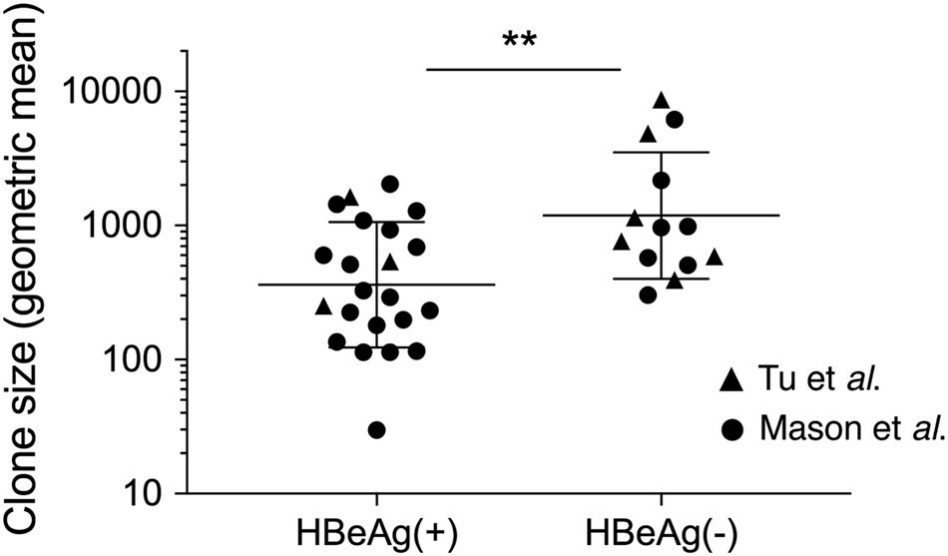
Clone sizes of hepatocytes containing virus-cell junctions in in vivo datasets. Hepatocyte clones detected in HBeAg-positive (*n* = 22) and HBeAg-negative (*n* = 13) groups in Tu et al.^18^ and Mason et al.^17,19^ were estimated by copy number of repeated virus-cell junctions contained in these cellular clones. Only accurately determined clone sizes using repeated virus-cell junctions detected in liver fragments (and not from liver slide sections or laser-microdissected material) were included for this comparison. Geometric mean ± SD; **p < 0.01, two-sided Mann–Whitney test

The HBV DNA integration events in this study were all detected using inverse nested PCR (invPCR), a sensitive method used to detect the right side of the integrated HBV dslDNA^16^ and downstream host cellular DNA sequence. We separated virus-cell junctions previously detected by invPCR^17–19^ into two groups: those found in HBeAg-positive patients and those found in HBeAg-negative patients (patient clinical details are summarized in Table S1). Importantly, these HBV DNA integrations have been detected in the non-tumor tissue of HBV patients, the majority of whom have no concurrent HCC. The clone sizes of hepatocytes in these patient cohorts (calculated with the copy number of repeated virus-cell junctions contained in these cellular clones) are shown in Fig. 1. As previously reported^17–19^, significantly larger hepatocyte clones are observed in HBeAg-negative patients compared to HBeAg-positive patients. This clonal expansion is likely driven by liver turnover following the activation of antiviral immune responses associated with HBeAg-seroconversion. These represented the main experimental comparison groups in our analyses.

As a control for the underlying biological processes of HBV DNA integration (e.g., lower integration rates in genes essential for cell survival), we used a dataset of HBV DNA integrations generated from a newly developed in vitro infection model^14^. Briefly, Huh7-NTCP cells were infected with 500 VGE per cell in a 24-well plate. After 1 day of inoculation, cells were washed and cultured for 3–7 days. Then, infected cells were transferred to a 12-well plate to induce a single round of mitosis, leading to a decrease in HBV replicative intermediate DNA. After 2 days, total DNA was isolated from expanded cells and analyzed by invPCR. The low level of expansion and selection in this system allowed this dataset to act as a control for HBV DNA integrations that are not yet selected for the microenvironment of the HBV-infected liver.

Further, we posited that the use of restriction enzymes in the invPCR process to excise the virus-cell DNA junction could potentially introduce a bias in favor of isolating integration events closer to the restriction sites in the host genome. Therefore, we developed an in silico model to control for the restriction site biases within the genome of the invPCR assay^14^. Briefly, we randomly simulated virus integration over the entire human genome and then filtered out those virus-cell junctions that did not pass our applied detection criteria (a more detailed description can be found in the methods section and in the ref. ^14^).

**Fig. 2:**
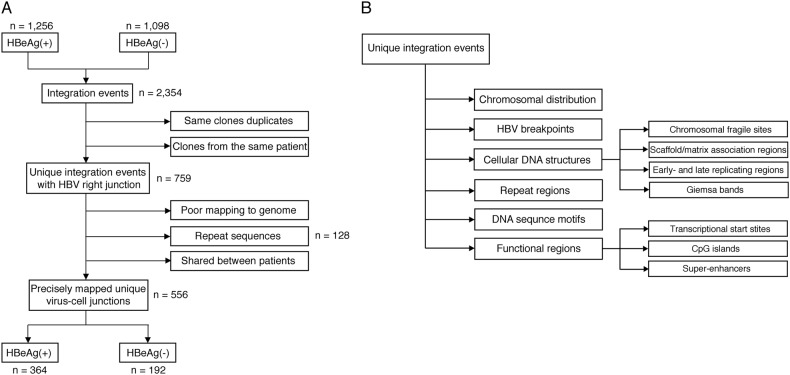
Analysis workflow. Flowcharts describing the analysis workflow to identify unique virus-cell junctions in HBeAg-positive and HBeAg-negative patients (**a**), and integration in the proximity of various cellular genomic features, including structural and functional regions (**b**)

The virus-cell junctions detected in these previous studies were subject to conservative inclusion criteria and aligned to the latest assembly of the human genome (hg38) (Fig. 2a). Final numbers of analyzed integration junctions are summarized in Table 1. Then, these datasets were analyzed for enrichment with respect to structural and functional features in the viral and human genomes (Fig. 2b).

**Table 1:** Patient numbers per group in the datasets used in this study

Publication	In silico	In vitro	HBeAg(+)	HBeAg(−)
Tu et al.^14^	10,000^a^	15^b^	0	0
Mason et al.^19^	–	–	19	7
Tu et al.^18^	–	–	3	10
Mason et al.^17^	–	–	0	5
Number of unique integration events after filtration	883	161	364	192

^a^Independent integrations generated in in silico simulation prior to filtering

^b^Independent infections

*HBeAg(+)* HBeAg-positive, *HBeAg(−)* HBeAg-negative

### Chromosomal distribution of integrated HBV does not change over the course of HBV infection

**Fig. 3:**
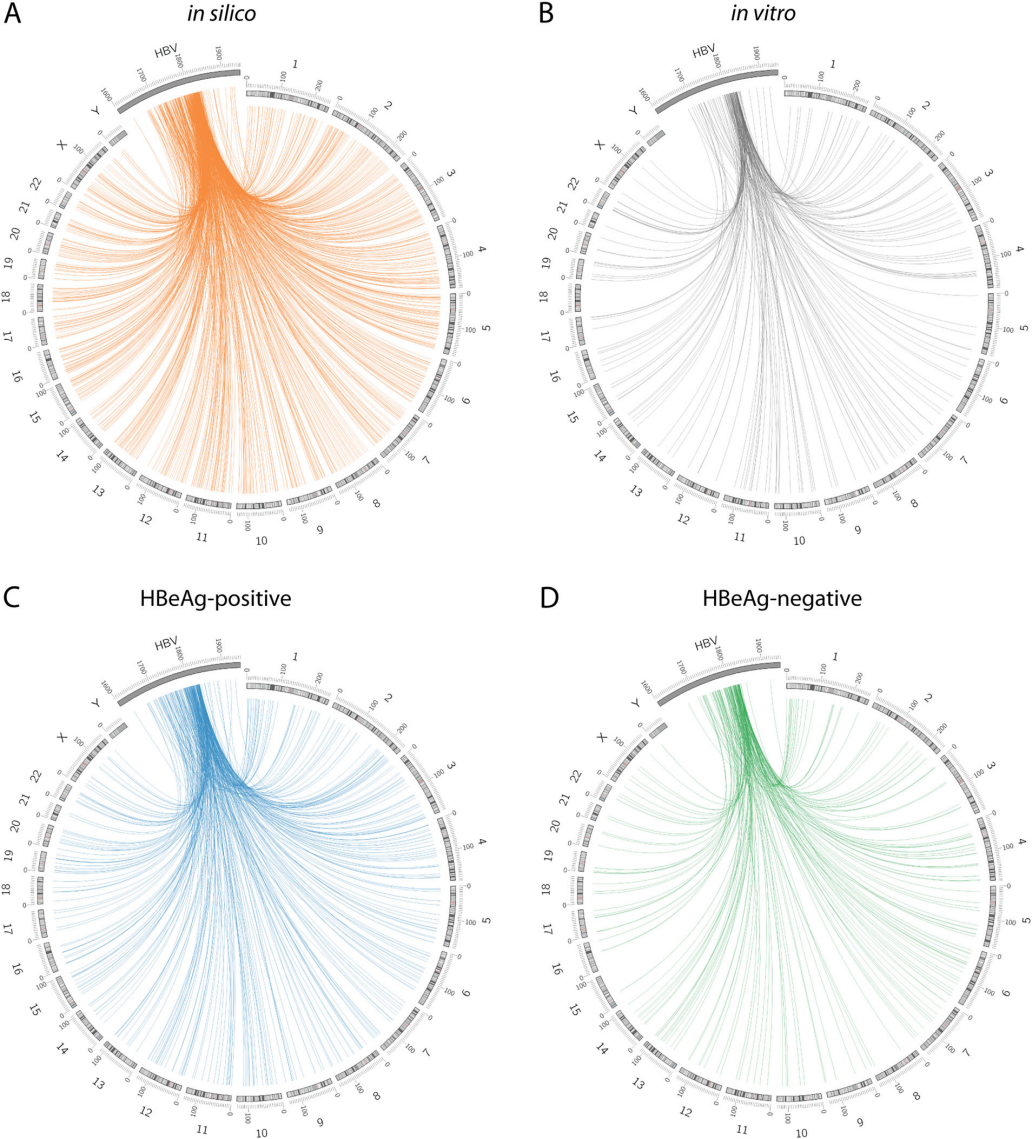
Chromosomal distribution of virus-cell junctions. Distribution of the integration breakpoints across human chromosomes and in the HBV genome. Each line represents an integration event at a particular locus in the HBV and human genome (hg38) in the in silico dataset (**a**), in vitro dataset (**b**), HBeAg-positive patients (**c**), and HBeAg-negative patients (**d**). Chromosome numbers are shown on the outer rim. Viral integration breakpoints were randomly produced in the in silico model based on the frequency distribution of HBV junctions observed in the in vitro and in vivo datasets. It should be noted that the HBV genome has been expanded in scale and cropped to the area analyzed by invPCR to show more detailed positional information

The distribution of HBV DNA integrations was first mapped with regard to the cellular chromosomes (Fig. 3). As expected from previous studies, the majority of integration events were distributed throughout the whole genome without any obvious chromosome preferential integration hotspots. After normalization of the number of integrations per dataset to the length of each chromosome, *Z*-score analyses of integration frequency in each of the chromosomes revealed little difference between the in vitro, HBeAg-positive, and HBeAg-negative datasets (Supplementary Figure 1).

Then, we mapped the HBV integration sites with respect to the virus genome (Fig. 3, Supplementary Figure 2). The majority of integration events (88.4, 90.1, and 93.8% for in vitro, HBeAg-positive and HBeAg-negative, respectively) occurred between HBV nucleotide positions 1700 and 1830, as previously reported^17–19^. These findings were consistent with the results of the WGS studies of tumor and matched adjacent non-tumor samples^6,9,22^ and indicate that HBV dslDNA is the main substrate of HBV integration. Further, no significant differences were observed between the three biological datasets, suggesting that particular HBV breakpoints are not selected during disease progression.

### HBV DNA integration is not enriched in specific cellular DNA structural regions during HBeAg-seroconversion

**Fig. 4:**
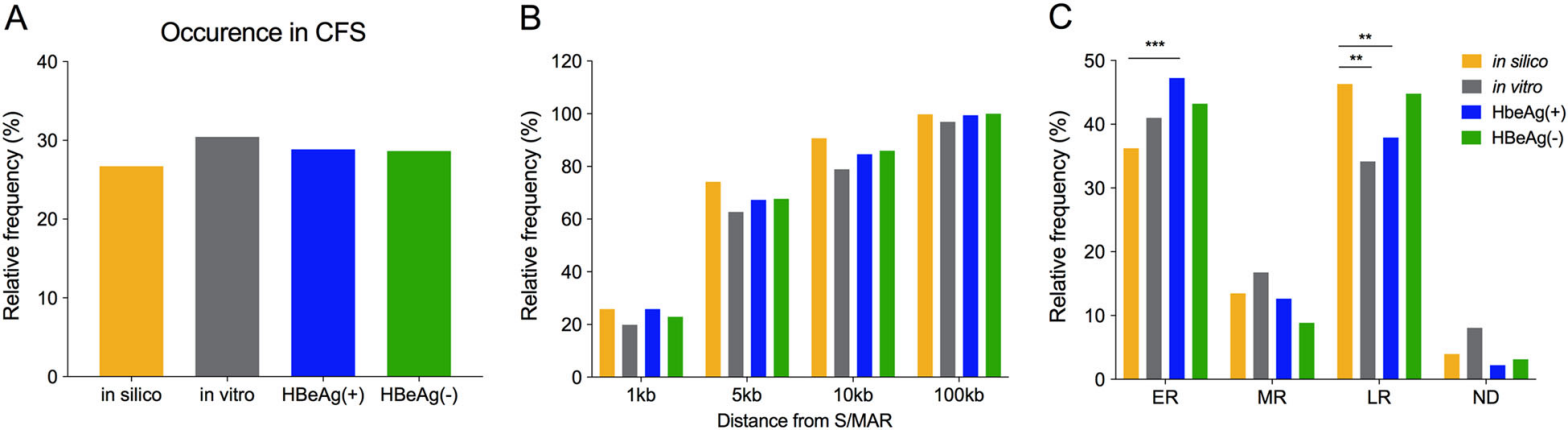
Cellular structural features in proximity to HBV DNA integration junctions. Percentages of HBV integration junctions in each dataset [in silico (gold), in vitro (gray), HBeAg-positive (blue), and HBeAg-negative (green)] were calculated with respect to (**a**) occurrence in chromosomal fragile sites (CFS), **b** proximity to S/MAR, and **c** occurrence in early-/late-replication timing regions (early replication (ER), mid replication (MR) and late replication (LR) regions of the host cell genome, and not distinguished (ND)). **p < 0.01 and ***p < 0.001, Normal approximation *z*-test

Next, we examined whether HBV preferentially integrates in proximity to specific genomic structural features. Here, we focused on genomic features previously reported to be associated with carcinogenesis, including integration into chromosomal fragile sites (CFS), scaffold/matrix attachment regions (S/MAR), and DNA regions of early and late replication timing (Fig. 4).

#### Chromosomal fragile sites (CFS)

First, HBV integration sites were assessed for preferential integration into CFS. CFS are highly susceptible to genomic changes, such as double-stranded DNA breaks during the replication stress, DNA rearrangements, DNA deletion, and DNA recombination^23–25^. HBV integrations in CFS have been reported to induce genetic instability, alter gene expression of miRNA and tumor suppressor genes and thereby play an important role in hepatocarcinogenesis^26^. In our datasets, 26.7, 30.4, 28.6, and 28.4% of HBV integration sites were observed in CFS in the in silico, in vitro, HBeAg-positive and HBeAg-negative groups, respectively (Fig. 4A, Supplementary Table 2), suggesting that HBV integrations were not significantly enriched in CFS regions.

#### Scaffold/matrix attachment regions (S/MAR)

Host S/MAR interactions with the nuclear scaffold and HBV integration events in these sites have been reported in the HCC tissue of animals with chronic woodchuck HBV (WHV) infection^27^ and human HCC cell lines^28^ and have been suggested to promote carcinogenesis via dysregulation of cellular oncoproteins^4,29^. In our analyses, we found that a large number of HBV integrations occurred in the proximity (within 5 kb) of predicted S/MAR (60–70%), though this did not significantly differ from our in silico control dataset. This suggests that the reported enrichment in previous studies may simply be due to the ubiquity of S/MAR in the cellular genome, rather than any selection pressure or intrinsic preference for HBV DNA to integrate in the proximity of these sites.

#### Early and late replicating regions

Recent studies have shown a correlation between replication timing and distribution of somatic mutations and copy number alterations in cancers with increased mutation frequency^30^. Therefore, we used Repli-seq data from a previous study^31^ to determine whether HBV integration was more likely to integrate in early or late-regions of replication (Fig. 4c). We found that in comparison to the in silico control, integrations of in vitro and in vivo datasets were slightly enriched in early replicating sites and slightly less likely to occur in late replicating sites.

In summary, HBV DNA integration into specific cellular DNA structural regions does not appear to be selected during HBeAg-seroconversion. Further, any observed enrichment into particular structural sequences (e.g., early replicating regions) appear to be intrinsic to the molecular mechanisms of HBV DNA integration itself, as they are also seen in the in vitro datasets.

### Specific DNA sequences are not enriched at the site of HBV integration

HBV integration events in specific sequences (e.g., repeat regions) have been suggested as potential drivers of the carcinogenic process. Previous next-generation sequencing (NGS) studies in tumor tissue have shown that >50% of HBV integration events occur in repeat regions^8^, and in some cases, reportedly generate pro-oncogenic fusion sequences with LINE-1^32^ or *Alu*^33^ transposable elements. Therefore, we used RepeatMasker^34^ to determine the proportion of HBV DNA integrations that occur in repeat regions but failed to observe differences between the biological and in silico datasets (Table 2).

**Table 2:** HBV DNA integration into cellular repeat regions

Type	In silico	In vitro	HBeAg(+)	HBeAg(−)
Tandem repeats	0.49	2.31	0.90	0.86
DNA transposons	2.25	3.24	2.26	2.16
*Retrotransposons*				
LTR	6.15	6.94	9.05	7.33
SINE	5.76	6.48	7.69	6.90
LINE	16.60	19.44	11.09	15.09
SVA	–	0.46	0.23	–
Others	0.88	1.39	2.94	0.86

All figures are given in percentages of the total number of integrations (after quality control filtration as shown in Fig. 2a) for each dataset

*HBeAg(+)* HBeAg-positive, *HBeAg(−)* HBeAg-negative, *LTR* long terminal repeat, *SINE* short interspersed nuclear elements, *LINE* long interspersed nuclear elements, *SVA* SINE-VNTR-Alu

We further examined potential enrichment of sequence motifs using MEME Suite^35^ to analyze the cellular genome sequences within 20 bp of either side of the HBV DNA integration junctions. As expected from random DNA integration, no enrichment for specific sequence motifs was observed at a rate greater than predicted by our in silico model (Supplementary Figure 3).

Due to the broad distribution of HBV integrations over the cellular genome^13^, the repair mechanism underlying HBV integration was assumed to be NHEJ at the site of double-stranded cellular DNA breaks. However, we and others have previously reported MMEJ as a repair mechanism in some instances^14,22^, as 2–6 bp of homology are observed at some virus-cell junctions. MMEJ has previously been associated with cancer initiation due to mutation generation through error-prone DNA repair; therefore, we investigated whether MMEJ-associated HBV junctions were enriched during HBeAg-seroconversion.

To determine the expected homologous sequence distribution, viral integration breakpoints were randomly produced in the in silico model based on the frequency distribution of HBV junctions observed in the in vitro and in vivo datasets, as previously described^14^. We found that there was no significant difference in the proportion of virus-cell junctions showing sequence microhomology in the in vitro, HBeAg-positive and HBeAg-negative datasets (Supplementary Figure 4).

### HBV DNA integration with respect to functional regions in the cellular genome

**Fig. 5:**
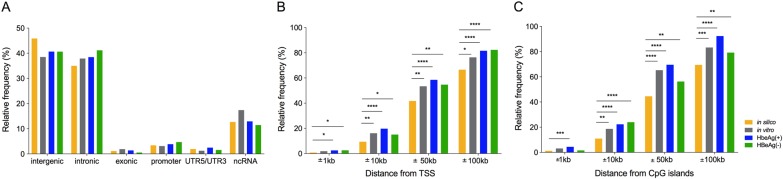
Cellular functional features in proximity to HBV DNA integration junctions. Percentages of HBV integration junctions in each dataset [in silico (gold), in vitro(gray), HBeAg-positive (blue), and HBeAg-negative (green)] were calculated with respect to occurrence in functional regions [separated into intergenic, intronic, exonic regions, promoters, UTRs and non-coding RNAs (ncRNA) (**a**)]. We also measured the distance from the transcriptional start site (TSS) of the closest gene (**b**) and the nearest CpG island (both upstream and downstream) (**c**). The frequency is shown as a percentage of all integration events per dataset. *p < 0.05, **p < 0.01, ***p < 0.001 and ****p < 0.0001, Normal approximation *z*-test

Finally, we determined whether HBV integrations were associated with specific functional regions in the cellular genome (Fig. 5). First, we measured the proportion of integrations that occur in coding and non-coding cellular regions (Fig. 5a). We found that 45.1, 46.6, and 47.4 of integrations were located in the genic region [which included exons, introns and regions <5 kb upstream of transcriptional start sites (TSS)] in the in vitro, HBeAg-positive and HBeAg-negative patient datasets, respectively, all of which were slightly enriched compared to the in silico integration model (41.4%). HBV DNA integrations into the host exonic regions were rarely detected: three genes contained HBV integrations in the in vitro dataset (ACTN3, COG8 and NUP2210L), 5 genes in the HBeAg-positive group (GLYATL1, RNF25, PCDHGB3, A1BG and HDHD3), and 1 in the HBeAg-negative group (IFNLR1). Therefore, we focused on the other genic regions.

Previous studies have shown that HBV integration occurs preferentially close to TSS in HCC tissue^22^. Therefore, we calculated the distance between every virus-cell junction and the nearest TSS for each dataset (Fig. 5b). Slight enrichment for HBV integration sites within <10 kb of a TSS was observed compared to our in silico control dataset, but no association was found with disease progression. This region around the TSS may include regulatory regions such as CpG islands, enhancers, and promoters. As reported above, integration into promoter regions is rare (Fig. 5a), and thus, we concentrated on CpG islands and enhancer regions.

We analyzed the proximity of HBV DNA integrations to the closest CpG island in all datasets. We found a slight (~2-fold) but highly significant enrichment within 10, 50, and 100 kb of CpG islands in all biological datasets compared to the control in silico dataset (Fig. 5c). This enrichment was seen both upstream and downstream of CpG islands (Supplementary figure 5).

**Fig. 6:**
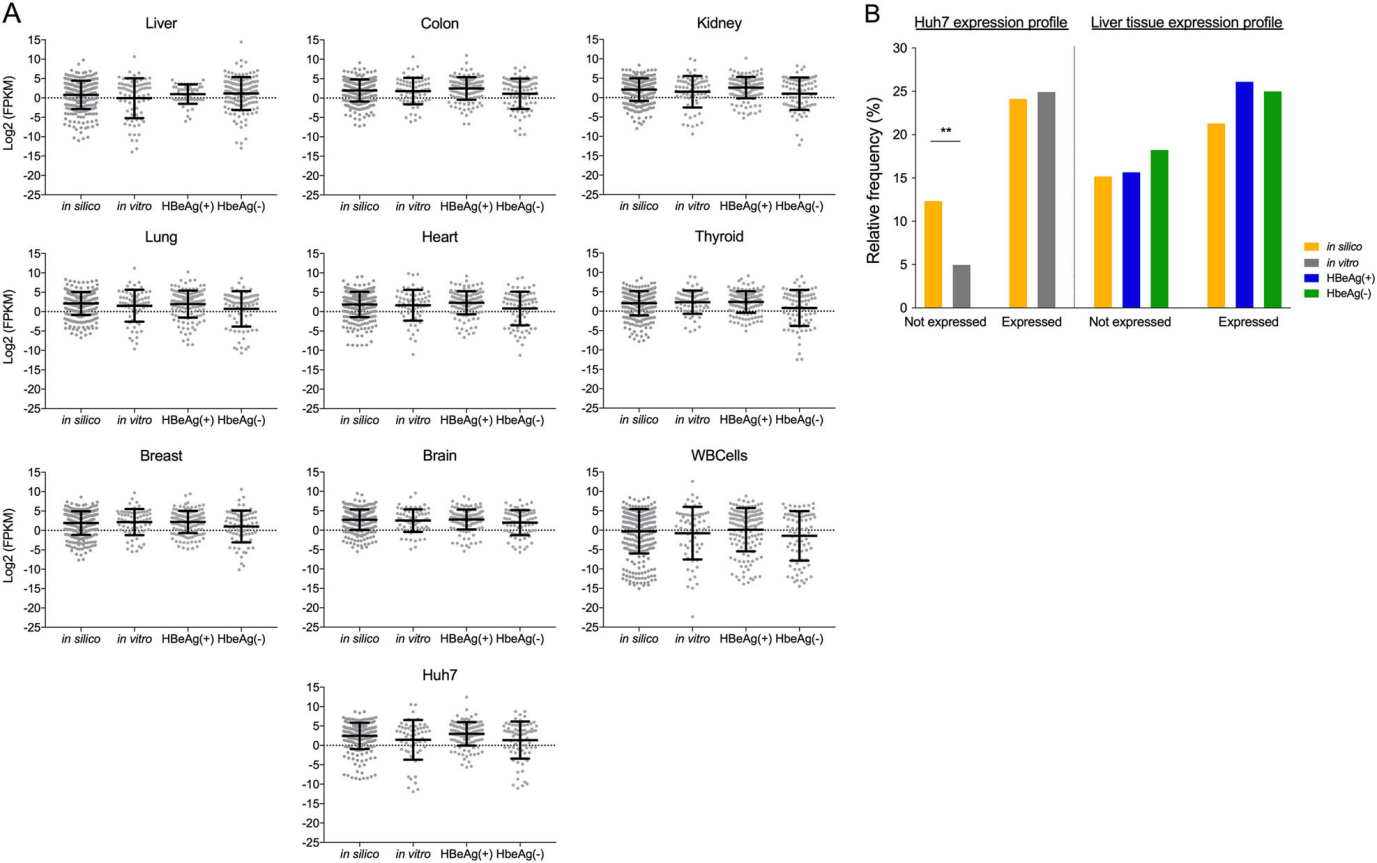
Tissue expression of genes containing HBV DNA integrations. Tissue transcriptional expression levels [mean fragments per kilobase per million mapped reads (FPKM) ± SD] of genes containing HBV DNA integration is shown for 9 normal tissues (liver, kidney, lung, colon, thyroid, breast, brain, heart and white blood cells) and Huh7 cells infected with HBV (**a**). Outliers were excluded using Robust regression and Outlier removal (ROUT) method. Percentages of HBV integrations in genes that were either expressed or not expressed in Huh7 (in silico and in vitro datasets) and liver tissue (in silico and in vivo datasets) (**b**). **p < 0.01, Normal approximation *z*-test

Then, we reasoned that if HBV integration was affecting cell phenotype (particularly due to insertional mutagenesis), these events would preferentially occur in expressed genes. Therefore, we analyzed the RNA-seq data of 9 different tissues (including liver) and Huh7, a human hepatoma cell line expressing HBV. The mean expression levels of genes in which integration events occurred in the in vitro and in vivo groups were not statistically significant compared to those in the in silico group (Fig. 6a). Indeed, the gene expression profiles of genes in proximity to integrations appeared identical regardless of the tissue source of the RNA-seq data, suggesting no association between HBV DNA integration and gene expression. We further studied the percentage of all integrations in genes that were either expressed or not expressed in either Huh7 cells (in silico and in vitro datasets) or liver tissue (in silico and in vivo datasets) (Fig. 6b). In most instances, we found no significant difference between in silico groups and the biological groups. Interestingly, we found a significantly lower proportion of integrations in non-expressed genes in the in vitro group compared to in silico control.

Finally, when genes proximal to HBV DNA integrations were analyzed for biological pathways using Ingenuity Pathway Analysis (IPA, Ingenuity Systems, Mountain View, CA; http://www.ingenuity.com), no specific cell functions were enriched in the in vitro and in vivo datasets compared to the in silico dataset.

### HBV integrations in recurrent or cancer-related genes are not enriched with disease progression

A number of studies have identified recurrent genes targeted by HBV integration that can potentially induce hepatocarcinogenesis, including telomerase reverse transcriptase (TERT) and mixed-lineage leukemia 4 (MLL4)^5–7,9^. Considering only integrations in coding, UTR, and promoter regions, we found recurrent integrations in 1 gene out of 71 integrations in the in vitro group (PKM, intronic), 4 out of 168 in the HBeAg-positive group (GRAMD4, MNX1, RGS1 and SPATA4, all intronic, shared in 2 patients), and 1 out of 92 in HBeAg-negative (NTN1, intronic). Given 20,376 coding genes in the cellular genome and using birthday problem probability analysis, we found the likelihood of this recurrence to be *p* = 0.11, 0.064, and 0.186 for the in vitro, HBeAg-positive and HBeAg-negative groups, respectively. This suggests that the incidence of recurrent integrations was not significantly above chance.

Of the integrations in genic regions found in in vitro, HBeAg-positive, and HBeAg-negative groups, 2, 11, and 4 integrations were found to reside within known oncogenes and 8, 11, and 7 integrations in tumor suppressor genes, respectively (Supplementary Table 3). Integrations into genes specifically associated with HCC (COSMIC database) were only observed in in vitro (CARS, ERBB4 and KDR; 1.9% of integrations in genic regions of the host genome) and HBeAg-positive groups (EGFR, MAP2K7, and TGFBR2; 1.1%), suggesting no significant enrichment with clonal expansion or HBV disease progression. Interestingly, only 0.45% of in silico integrations in genic regions fall into HCC-associated genes, which may suggest slight enrichment in these genes in biological samples.

## Discussion

After analysis of 717 unique virus-cell junctions detected in an in vitro culture system and in patient tissues (one of the largest analyses of HBV DNA integrations in non-tumor tissue) with comparison to an in silico-generated control dataset, we observed no obvious enrichment for specific structural or functional genomic features during HBeAg-seroconversion. No specific enrichment in the cellular sequences adjacent to the HBV DNA integration sites were observed, including: specific cellular chromosomes; chromosomal fragile sites; intergenic/intronic/exonic regions; enhancer regions; CpG islands; transcriptional start sites; and transcriptional activity of the regions. While random chromosomal distribution and lack of enrichment of HBV DNA integration into specific functional genomic regions have been previously reported^7,22^, we show that the sites of HBV DNA integration are not under strong selection during the major clonal expansion phase of disease progression in chronic HBV infection. This is particularly evident when considering that the HBV DNA integration profile in the in vitro infection dataset is almost identical to those in the in vivo datasets, despite a >1000-fold clonal expansion in the latter. In light of this data, the hypothesis that HBV integration initiates a carcinogenic phenotype (prior to hepatocarcinogenesis) via insertional mutagenesis or *cis*-regulation of cellular genes is poorly supported.

The most likely explanation for our results is that the majority of HBV DNA integrations act as passenger mutations and do not represent HCC driver events or initiators of carcinogenesis. Indeed, the majority of DNA mutations leading up to HCC appear to be passenger mutations^36^. This contrasts with previous NGS studies showing an enriched integration in HCC-associated genes and other structural and functional regions of the cellular genome^4–7,9^. In these previous reports, weak enrichment (though statistically significant) towards cancer-associated genes or DNA regions was not present in the majority of tissue samples or the majority of HBV integrations, suggesting that these are likely to be rare phenomena. We believe that many of these reports of HBV DNA integration into specific cellular regions may have been falsely interpreted as initiators of hepatocarcinogenesis due to low numbers of HBV DNA integration events analyzed and the lack of strong controls (e.g., detection bias and intrinsic molecular preferences of HBV DNA integration), which we have addressed in this paper. Based on the data presented here, integrated HBV DNA may be acting on cell phenotype through *cis*-mediated mechanisms during cancer progression as opposed to cancer initiation.

There are other reported pro-oncogenic mechanisms (particularly those that are driven by HBV DNA integration *in trans*) that cannot be addressed by our approach, such as increased overall genomic instability due to HBV integration^26^ or the expression of HBV antigens (either wild-type or mutant) from integrated HBV DNA^37^. The contribution of these mechanisms to HCC initiation in HBV infection is still unknown and outside the scope of this study. However, other data from the field would suggest that HBV DNA integration *per se* does not drive the initiation of HBV-associated HCC through these mechanisms (described below).

First, HBV-associated HCCs with detectable HBV integrations do not appear to have extensively different genomic or mutational profiles compared to those of HCCs without HBV integrations, suggesting that genomic instability is not generally driven by HBV integration. Whole genome HCC analysis of HBV patients show that large chromosomal rearrangements were reportedly enriched compared to HCCs of other aetiologies^38^. However, it is not clear if these rearrangements are caused by p53 mutations or known concurrent exposure to aflatoxin (both are known to contribute to chromosomal instability^39^) enriched in these reported cohorts rather than due to HBV infection itself. Further, HBV DNA integrations have been associated with areas in the human genome that have higher somatic mutations and greater chromosomal copy number variations^22^. However, these DNA lesion-rich areas could exist prior to integration and simply have a greater incidence of double-stranded DNA breaks (the substrate for HBV integration), instead of being directly caused by HBV integration.

In addition, high expression of HBV antigens occurs during the immune tolerance phase when HCC risk is low in humans, suggesting HBV antigen expression itself cannot initiate frank cancer. Whether HBV expression contributes to the carcinogenic process in the presence of other carcinogens is still a controversial point; for example, the majority of studies of transgenic mice expressing HBV antigens under native promoters have reported close to normal HCC risk in these animals^40–42^, though some have reported that they are highly susceptible to spontaneous HCC formation^43^. Thus, it is unclear if chronic expression of HBV antigens from integrated HBV DNA per se drives the initiation of HCC.

The strength with which we can assert these hypotheses is constrained by drawbacks in our approach, including the detection of only a subset of all HBV DNA integrations using the invPCR assay. While we have controlled for biases towards integration events occurring in proximity to the necessary restriction sites using our in silico model, the invPCR assay is limited in detecting integrations that occur between nucleotides ~1650 and ~1850 (with respect to the HBV genome). The 3′ terminus of the HBV dslDNA has been observed at the junction by unbiased whole genome sequencing analyses for the majority of HBV DNA integrations, though a significant fraction of integrations have junctions outside this area^44^. It is possible that these other forms of HBV DNA integrations may be causing rare insertional mutagenesis events, but integrations occurring near the 3′ terminus of HBV dslDNA have been reported as the main supposed drivers of hepatocarcinogenesis through the formation of HBx-cellular fusion transcripts and driving downstream cellular genes through the exposed HBV core promoter. We find evidence of neither mechanism driving the significant clonal expansion of hepatocyte clones in pre-neoplastic tissue.

Despite these shortcomings, our sequence analysis can be used to speculate the underlying biological processes of HBV DNA integration. We found that the cellular sites directly up or down-stream of HBV DNA integrations were not enriched for specific sequence motifs at a rate greater than chance; however, there was a slight enrichment (~2-fold over the in silico dataset) of integrations within 10 kb of CpG islands in both in vitro and in vivo datasets. Enriched integration in CpG islands has been described in prior studies in HCC tumors^22^, but here, we are the first to describe it in non-tumor tissue and in in vitro HBV infection models. Our data suggests that the molecular mechanism of HBV integration intrinsically directs integration in proximity to CpG islands (or TSS), rather than specifically causing pre-neoplastic changes that then undergo positive selection. Possibly, open chromatin is more susceptible to breaks and therefore integration, as we found significantly fewer integrations within non-expressed genes in our in vitro dataset (Fig. 6b). However, the exact biological mechanism behind this phenomenon is not clear and requires further experimental work in our in vitro integration model.

Finally, we found that HBV DNA integrations generated by in vitro infection do not significantly differ from those in the in vivo datasets. This suggests that our in vitro infection system is sufficient to recapitulate in vivo integration events accurately, despite taking place in a transformed cell line (Huh7). We speculate that in the population of “normal” HBV-infected hepatocytes, there may exist sub-populations of cells with defective/altered DNA repair mechanisms (e.g., hyper-activation of NHEJ pathways relative to homologous DNA repair, as seen in Huh7 cells^45^) in which integrations occur at higher rates. Clonal outgrowth of these cells (as observed in HBeAg-seroconversion) may increase the risk of HCC. In this hypothetical model, integrated HBV DNA is not the cause of preneoplastic changes but is a marker of them. Further research in our in vitro model is required to determine these details.

In summary, our study shows that the pre-neoplastic clonal expansion of hepatocytes is not associated with significant enrichment for HBV DNA integrations in specific functional sites in the human genome. Moreover, the underlying molecular process of HBV DNA integration appears to preferentially target sites proximal to cellular CpG islands and TSS through unknown mechanisms. Thus, our in-depth analysis of large datasets of virus-cell junctions with strict control datasets has shed new light on the reported mechanisms of HCC-initiation associated with HBV DNA integration.

## Materials and methods

### Datasets used

Four datasets of virus-cell junctions detected using inverse nested PCR method summarized in Table 2 were analyzed: three from previously published studies from primary liver tissues (*n* = 41)^17–19^ and one from an in vitro HBV infection model (GenBank Accession Numbers: MH057851-MH058006)^14^. All datasets analyzed during the current study are available from the corresponding author on reasonable request. Further, data from an in silico simulation was used as a control for detection biases in the invPCR assay^14^. In short, 10,000 genomic positions distributed randomly across the entire genome were selected using BEDTools (v2.26.0)^46^. Restriction enzyme cleavage sites for *Nco*I, *Bsi*HKAI and *Sph*I in the human reference genome GRCh38.p7 (hg38) were determined using Bowtie (v1.1.1)^47^. To filter out loci that would not be detected by invPCR, three selection criteria were applied: (1) the *Nco*I restriction enzyme site occurred <2 kb downstream of the junction (as invPCR products >2 kb were rarely detected in empirical studies); (2) the restriction enzymes *Bsi*HKAI and *Sph*I did not occur between the integration junction and the *Nco*I site; and (3) the sequence inserted between the junction and the *Nco*I site was longer than 20 nt to allow for accurate alignment to the human genome.

### Bioinformatic analysis

#### Alignment and annotation of functional and structural regions in the cellular genome

FASTA sequences were aligned against human (GRCh38.p7) and HBV genomes (GenBank accession numbers: AM282986 (genotype A), AB033554 (B), AB048704 (C), V01460 (D), AB032431 (E), X69798 (F)) using a standalone version of BLAST ncbi-blast-2.6.0+ (National Center for Biotechnology Information, Bethesda, MD, USA) with a custom created database. Then, we filtered sequences according to previously described exclusion criteria^14^. The position of the breakpoint was defined as the junction of HBV and host genome sequence. The repeat elements were annotated using Repeat Masker^46^ and sequences that fully fell into the repetitive regions were excluded. All integration breakpoints were annotated using ANNOVAR^48^. Numbering of the HBV nucleotides in all subsequent analyses is according to HBV ayw subtype (GenBank Accession #V01460.1^49^). Each HBV integration site was defined by its position on the cellular chromosome (hg38) and integration proximal genes were annotated based on the transcription start site from UCSC Genome Browser (http://genome.ucsc.edu/)^50^.

Association with genomic and chromosomal instability was assessed by enrichment analysis of HBV integration events in or near CFS, CpG islands, and S/MAR. Genomic locations of CFS were obtained from a manually curated list^51^, and CpG island coordinates from UCSC. Where required, data position coordinates were converted to hg38 reference genome coordinates using UCSC liftOver. S/MAR analysis applied to the 100 kb cellular DNA sequence surrounding the integration site using MARSCAN (EMBOS) algorithm (http://www.bioinformatics.nl/cgi-bin/emboss/marscan)^52^.

#### Replication timing

The replication timing (RT) of the regions that were affected by HBV integrations was analyzed using Repliscan, as previously described^53^. HepG2 cell line Repli-seq dataset (BAM files) containing samples from 6 fractions of S phase (G1b, S1, S2, S3, S4, and G2) was downloaded from ENCODE (GEO accession: GSM923446)^31^. As this dataset does not contain any non-replicating G1 control, we combined all S phases to use as a control. The Repliscan was run with two S-phase fractions: Early (early, early-mid) and Late (mid-late, late). Genomic windows (10 kb) were calculated using BEDTools across the whole genome and the data from each S-phase was divided by the control to normalize for sequence bias. Haar wavelet smoothing was performed to reduce the noise. The threshold signal of the RT was calculated and used to determine the predominant replication time in which a 10 kb window replicates. Next, signals were divided by the maximum value in the genomic window, resulting in the highest value being set as 1 and all other values between 0 and 1. A cut-off of >0.5 was used to classify a window as predominantly replicating. As this method allowed for a window to be predominantly replicating in more than one S phase (signals within 50% of the maximum value), the final classification includes four groups: early replication (ER), mid replication (MR), late replication (LR), and not distinguished (ND). Genomic locations of RT classes were converted from hg19 to hg38 coordinates using UCSC liftOver. The overlap of integration breakpoints with RT classes was determined using BEDTools intersect.

#### Transcription analysis

RNA-Seq data from nine different human tissues (liver, kidney, lung, colon, thyroid, breast, brain, heart, and white blood cells) from Illumina Human Body Map 2.0 Project (www.illumina.com; E-MTAB-513) was analyzed to assess the expression levels of the genes affected by HBV DNA integration. RNA-Seq sequences from Huh7 cells infected with HBV were downloaded from NCBI-Short Read Archive (SRA) (Bioproject PRJNA222881)^54^. STAR algorithm^55^ was used to align RNA-Seq reads to human reference genome hg38 (GRCh38.p7) followed by quantification of reads using Cufflinks^56^. Transcript expression levels of each gene were quantified in Fragments Per Kilobase per Million mapped reads (FPKM). Genes were classified into four categories based on the FPKM values: not expressed (0 to <1 reads), low (1–5 reads), medium (5–10 reads) and high (>10 reads) expression.

#### Analysis of biological pathways

The Ingenuity Pathway Analysis (IPA, Ingenuity Systems, Mountain View, CA; http://www.ingenuity.com) was used to identify the pathways and biological functions of genes affected by HBV DNA integration. The significance was set at a *p*-value of 0.01 by the right-tailed Fisher Exact Test.

The list of human tumor suppressor genes was taken from the Tumor Suppressor Gene Database (TSGene 2.0)^57^, oncogenes from ONGene database^58^, and a list of cancer and liver cancer mutated genes from the Catalog Of Somatic Mutations In Cancer (COSMIC v83)^59^.

### Statistical analysis

The statistical analyses were performed using GraphPad PRISM version 7.0b (GraphPad Software, La Jolla California USA). The differences between groups of discrete variables were analyzed using the Mann–Whitney test. A two-proportion *z*-test was used to assess the differences between proportions. A *p* < 0.05 was considered as being statistically significant.

## Electronic supplementary material

Supplementary Figure 1

Supplementary Figure 2

Supplementary Figure 3

Supplementary Figure 4

Supplementary Figure 5

Supplementary Table 1

Supplementary Table 2

Supplementary Table 3
