# Supplementary figures and images for: Sequence analysis of integrated hepatitis B virus DNA during HBeAg-seroconversion
Source: Emerg Microbes Infect. 2018 Aug 8;7:142. doi: 10.1038/s41426-018-0145-7 (PMC6081408; doi:10.1038/s41426-018-0145-7)

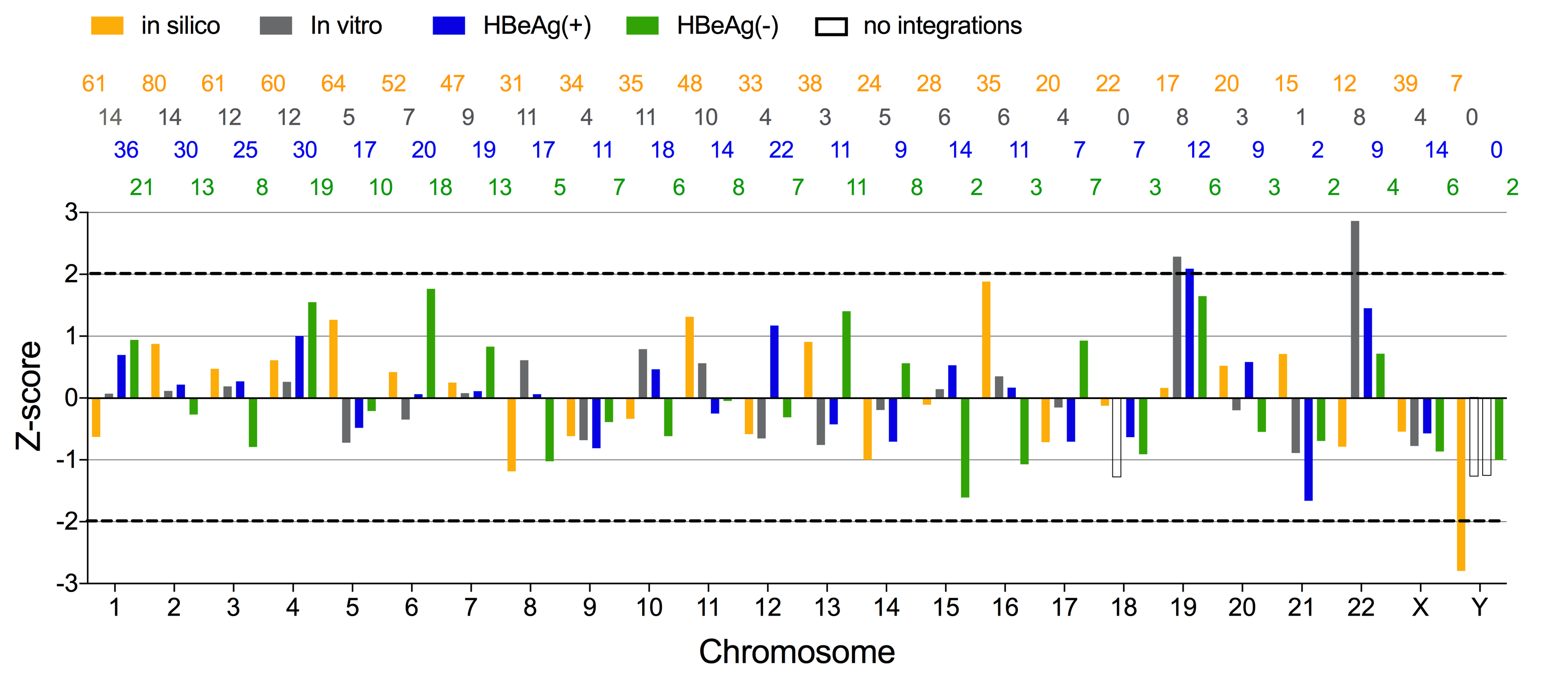

Supplement: Supplementary file 1 — Supplementary Figure 1 [file 41426_2018_145_MOESM1_ESM.tif]

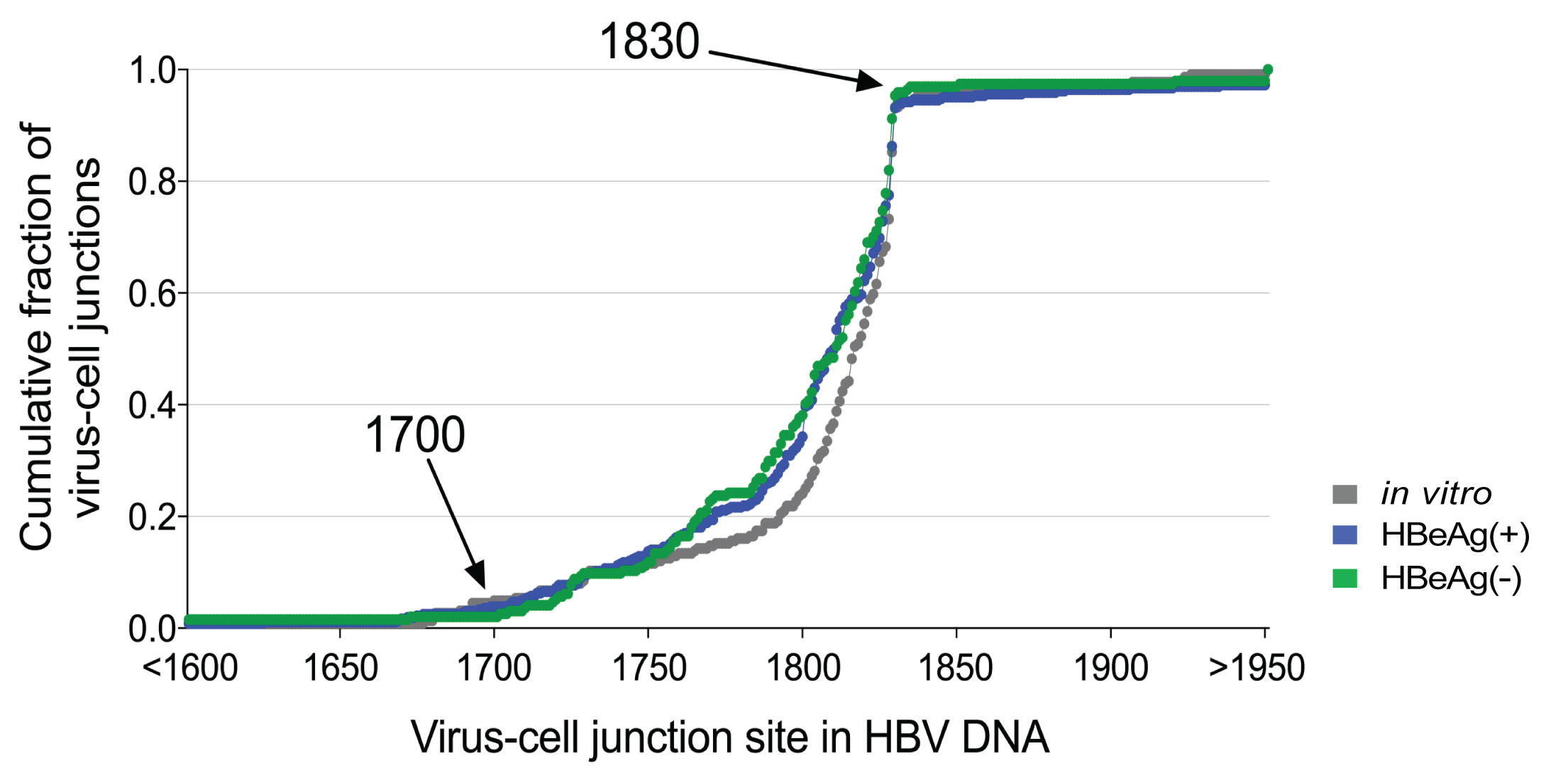

Supplement: Supplementary file 2 — Supplementary Figure 2 [file 41426_2018_145_MOESM2_ESM.tif]

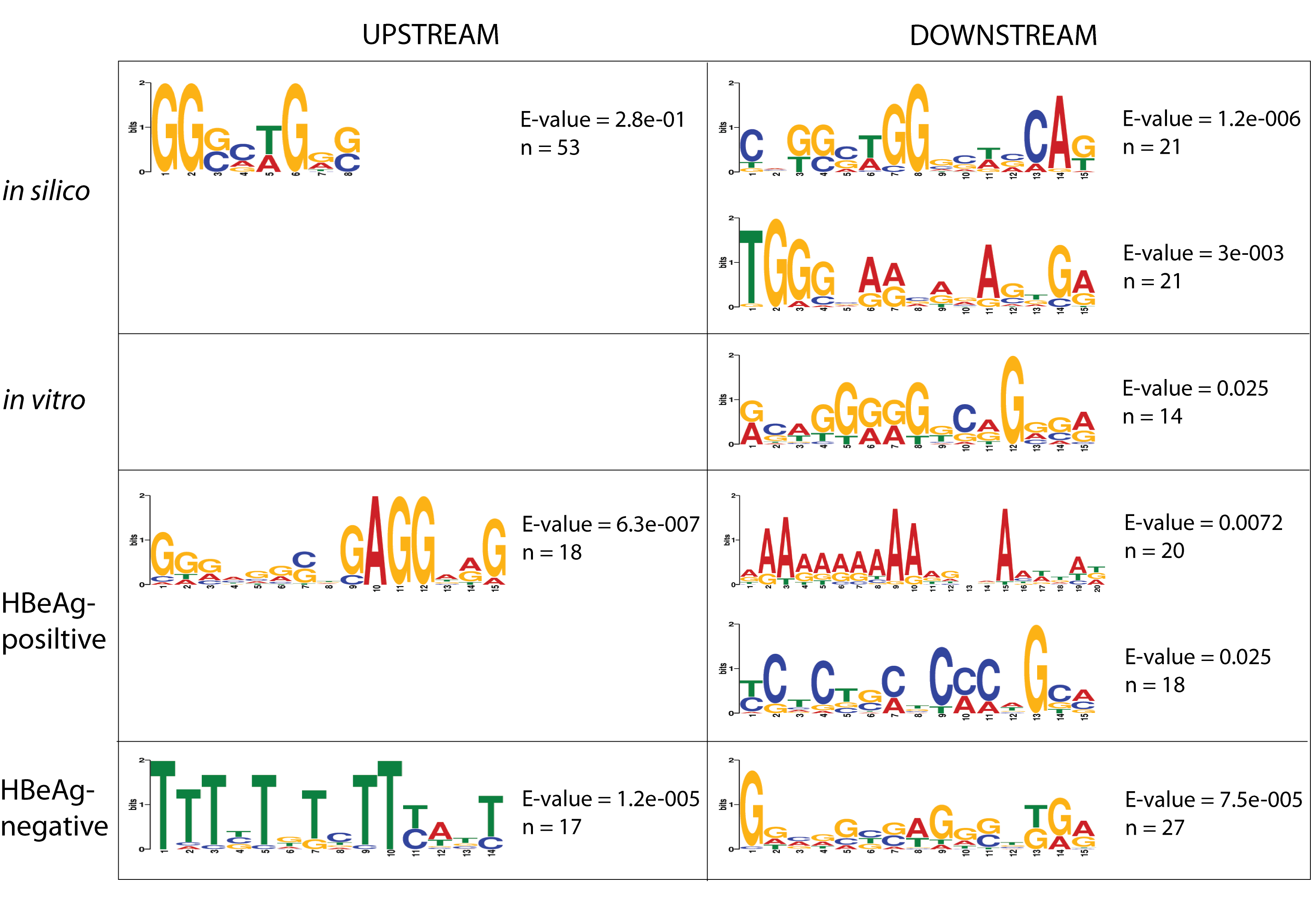

Supplement: Supplementary file 3 — Supplementary Figure 3 [file 41426_2018_145_MOESM3_ESM.tif]

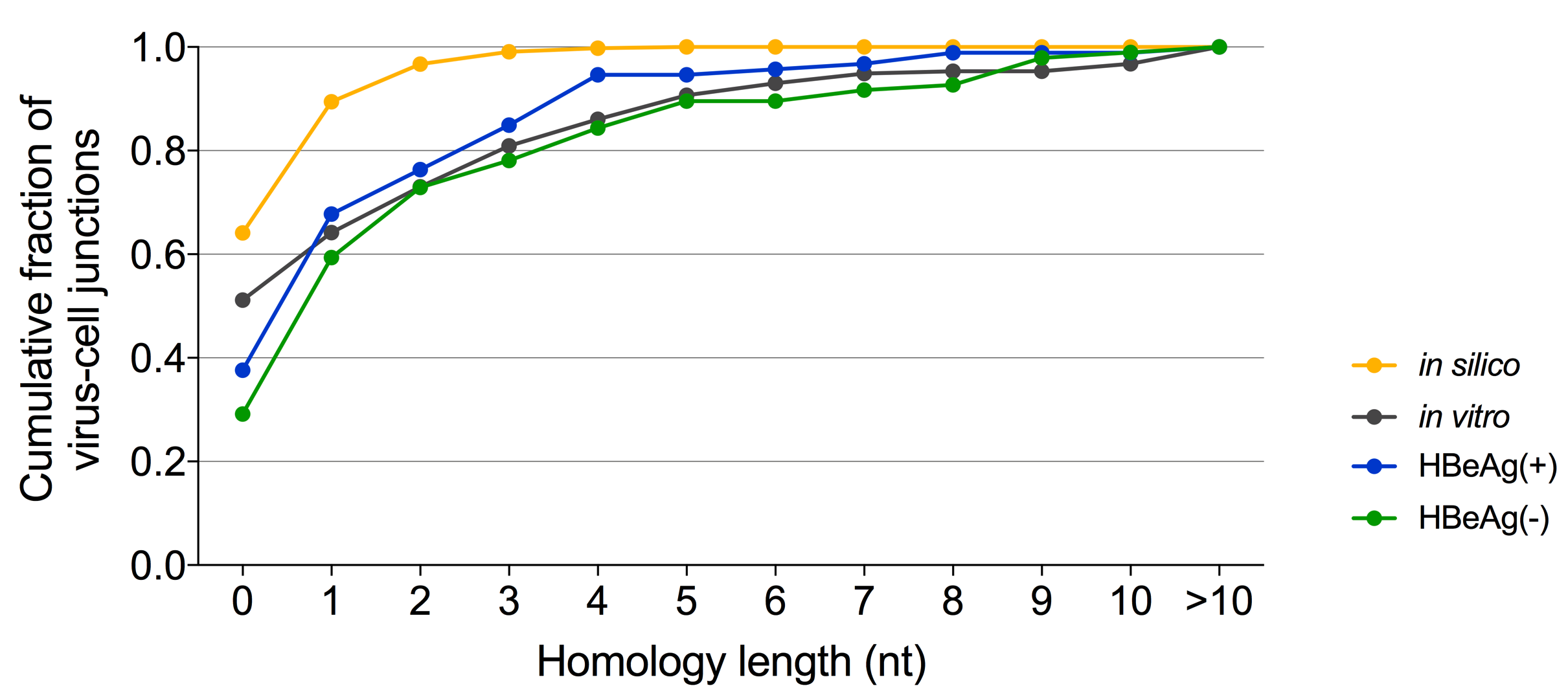

Supplement: Supplementary file 4 — Supplementary Figure 4 [file 41426_2018_145_MOESM4_ESM.tif]

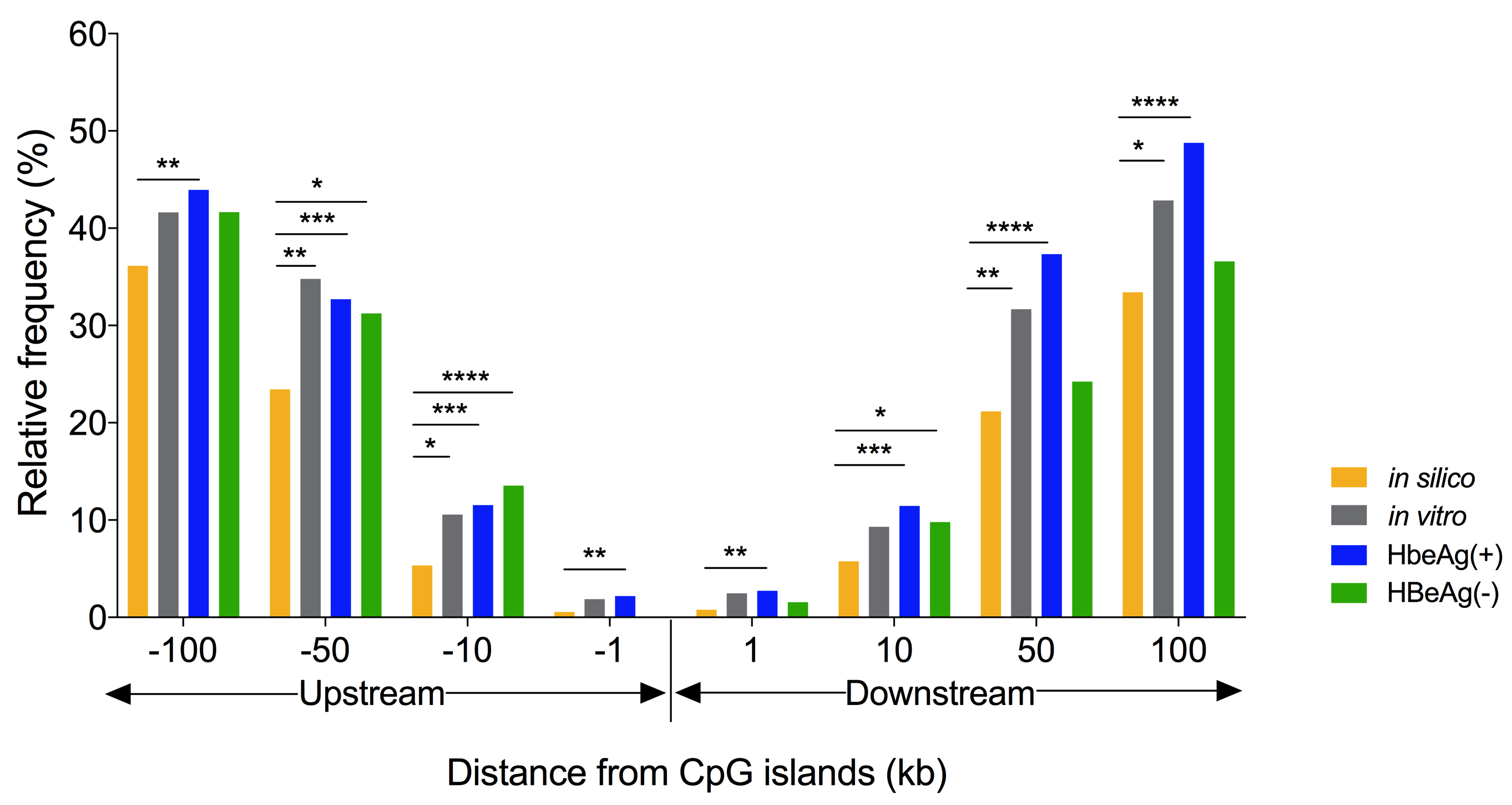

Supplement: Supplementary file 5 — Supplementary Figure 5 [file 41426_2018_145_MOESM5_ESM.tif]
