# Supplementary material for: Sequence analysis of integrated hepatitis B virus DNA during HBeAg-seroconversion
Source: Emerg Microbes Infect. 2018 Aug 8;7:142. doi: 10.1038/s41426-018-0145-7 (PMC6081408; doi:10.1038/s41426-018-0145-7)
Supplement: Supplementary file 6 — Supplementary Table 1 [file 41426_2018_145_MOESM6_ESM.docx]

### Table S1 – Clinical features of patients used in this study

| **Clinical features** | **HBeAg(+)**  **(n= 22)** | **HBeAg(-)**  **(n= 22)** |
| --- | --- | --- |
| Age (mean±SD) | 23.2±7.3 | 43.1±16.1 |
| Sex (M/F) | 10/12 | 19/3 |
| Viral genotype (A/B/C/D/E) | 3/4/6/6/3 | 3/4/7/7/1 |
| Fibrosis  (Ishak score 0/1/2/3/4/5/6) | 2/11/4/4/0/0/1 | 2/4/3/2/4/0/7 |
